# Adult mortality among second-generation immigrants in France: Results from a nationally representative record linkage study

**DOI:** 10.4054/demres.2019.40.54

**Published:** 2019-06-27

**Authors:** Michel Guillot, Myriam Khlat, Matthew Wallace

**Affiliations:** 1 Population Studies Center, University of Pennsylvania, Philadelphia, USA; 2 Institut National d’Études Demographiques (INED), Paris, France; 3 SUDA, Stockholm University, Stockholm, Sweden

## Abstract

**BACKGROUND:**

France has a large population of second-generation immigrants (i.e., native-born children of immigrants) who are known to experience important socioeconomic disparities by country of origin. The extent to which they also experience disparities in mortality, however, has not been previously examined.

**METHODS:**

We used a nationally representative sample of individuals 18 to 64 years old in 1999 with mortality follow-up via linked death records until 2010. We compared mortality levels for second-generation immigrants with their first-generation counterparts and with the reference (neither first- nor second-generation) population using mortality hazard ratios as well as probabilities of dying between age 18 and 65. We also adjusted hazard ratios using educational attainment reported at baseline.

**RESULTS:**

We found a large amount of excess mortality among second-generation males of North African origin compared to the reference population with no migrant background. This excess mortality was not present among second-generation males of southern European origin, for whom we instead found a mortality advantage, nor among North African–origin males of the first-generation. This excess mortality remained large and significant after adjusting for educational attainment.

**CONTRIBUTION:**

In these first estimates of mortality among second-generation immigrants in France, males of North African origin stood out as a subgroup experiencing a large amount of excess mortality. This finding adds a public health dimension to the various disadvantages already documented for this subgroup. Overall, our results highlight the importance of second-generation status as a significant and previously unknown source of health disparity in France.

## 1. Introduction

The native-born children of immigrants, also called second-generation immigrants, constitute a growing and increasingly diverse population in many countries of the European Union. In the EU as a whole, the population of second-generation immigrants with at least one foreign-born parent increased by 21.0% between 2008 and 2014, with larger increases (33.4%) for those of non-EU origin (Agafiţei and Ivan 2017). In proportionate terms, second-generation immigrants represented 6.0% of the total EU population in 2014, up from 5.2% in 2008 (Agafiţei and Ivan 2017). Although research is sparse, second-generation status has been identified in previous studies as an important source of health disparities in EU countries, with important disadvantages in mortality outcomes for certain second-generation subgroups, especially those of non-EU origin (Harding and Balarajan 1996; Razum et al. 1998; Tarnutzer, Bopp, and Grp 2012; Scott and Timæus 2013; De Grande et al. 2014; Manhica et al. 2015; Vandenheede et al. 2015; Wallace 2016; Jervelund et al. 2017).

Explanations for these mortality disadvantages include lower socioeconomic status, detrimental health behaviors, and chronic stress arising from perceived discrimination (Scott and Timæus 2013; De Grande et al. 2014; Manhica et al. 2015; Vandenheede et al. 2015; Wallace 2016; Jervelund et al. 2017). These patterns of excess mortality contrast with the situation of immigrants per se (i.e., the first generation) who tend to experience a mortality advantage despite lower socioeconomic status, a well-known paradox explained in part by migration selection effects (also referred to as the “healthy migrant effect”) (Razum et al. 1998; Palloni and Morenoff 2001; Bourbeau 2002; Khlat and Darmon 2003; Crimmins et al. 2005; Gushulak 2007; Riosmena, Wong, and Palloni 2013; Vang et al. 2017).

Among EU countries with populations greater than 1 million, France is the country with the largest second-generation population in both absolute and relative terms. In 2014, France’s population of second-generation immigrants with a least one foreign-born parent reached 9.5 million, representing 14.3% of the total population (Agafiţei and Ivan 2017). This high proportion is the product of France’s specific immigration history. Although not considered a ‘classic’ country of immigration, France stands out in Europe as the oldest country of immigration and the one that has received the largest cumulative number of immigrants. The earlier migration flows to France involved primarily immigrants from European countries (Italy, Spain, Portugal, Belgium, and Poland), followed after 1945 by large waves of ‘colonial’ migrants (mostly from North Africa). Despite a decrease in labor migration after 1973, immigration to France continued, mostly via family reunification, and the diversity of immigrants continued to increase, with larger proportions of immigrants from sub-Saharan Africa and Asia. This immigration history has generated a second-generation population that, today, is both large and diverse. The regions of origin most represented among second-generation immigrants are southern Europe (Portugal, Italy, or Spain) and North Africa (Algeria, Morocco, or Tunisia), which each region totaling about one-third. The last third comprises a very diverse set of parental countries of origin, including countries in sub-Saharan Africa, Europe, and Asia.

Previous studies have shown that in France, second-generation immigrants of non-EU origin, particularly those of North African origin, experience systematic disadvantages in important areas such as educational attainment, employment, and income (Silberman and Fournier 1999; Canaméro, Canceill, and Cloarec 2000; Meurs, Pailhé, and Simon 2006; INSEE 2012; Brinbaum, Primon, and Meurs 2016). The extent to which they also experience disadvantages in the area of mortality, however, has not been previously examined. This is a significant gap given the size of the second-generation population in France and the importance of documenting health disparities for informing evidence-based public health policies.

In this paper, we take advantage of a unique data source to estimate mortality by second-generation status in France. We focus on adults ages 18 to 64 and on the two main regions of origin of second-generation immigrants in the French context: southern Europe (Italy, Spain, and Portugal) and North Africa (Algeria, Morocco, and Tunisia). We compare adult mortality levels for second-generation immigrants with their first-generation counterparts and with the reference (neither first- nor second-generation) population. We also examine whether mortality differentials for second-generation adults remain after adjusting for educational attainment. To our knowledge, this is the first time that adult mortality patterns among second-generation immigrants in France are examined.

## 2. Methods

### 2.1 Data sources

The identification of second-generation immigrants in statistical sources is notoriously difficult as it requires information on parental place of birth, a variable that is rarely collected in surveys. In France, difficulties are compounded by the fact that a significant portion of the North Africa–born population is made up of ‘repatriates’ – a group of mostly European-origin individuals who were born in Algeria during the colonial period and relocated to France following Algeria’s independence in 1962 – rather than immigrants per se. This makes parental place of birth an insufficient variable for identifying second-generation immigrants of North African origin. Moreover, the French constitution prohibits the collection of information on ethnicity in official statistical sources, leaving few options for identifying second-generation immigrants, particularly those of North African origin (Simon 2015).

Nonetheless, we identified one data source that provides solutions to these identification issues while also containing mortality information. This source, called *Echantillon Longitudinal de Mortalité* (ELM; Longitudinal Mortality Sample), combines a baseline survey of the adult population living in France in 1999 with linked death records through 2010. The baseline 1999 survey, called *Etude de l’Histoire Familiale* (EHF; Family History Survey), is a random sample of approximately 380,000 individuals ages 18 and older who, as part of the 1999 census in France, were requested to fill in an additional questionnaire documenting their family history, including parental place of birth and languages used by parents to speak with the respondent when the respondent was age 5. The EHF information for these individuals was then matched, using identifying information on the respondent’s name, date, and place of birth, with France’s National Directory for the Identification of Natural Persons (RNIPP), an exhaustive population register that tracks identification information as well as civil status information of all residents of France. Survival status information (dead vs. alive) at the end of the observation period (15 April 2010), as well as the date of death for those who died during the observation period, was provided for the EHF individuals who were matched with the RNIPP. Information on causes of death was not included. The ELM did not track international out-migrations; individuals who were matched with the RNIPP but left France permanently during the period of observation thus appear in the ELM sample as ‘alive’ in 2010.

### 2.2 Study parameters

We focus on the two main regions of origin of immigrants and their native-born children in France: southern Europe (Italy, Portugal, and Spain) and North Africa (Algeria, Morocco, and Tunisia). First-generation (G1) immigrants were defined as individuals born abroad, and second-generation (G2) immigrants were defined as individuals born in France to two parents born abroad. We also identified individuals born in France to one parent born in France and one parent born abroad, called ‘mixed second generation’ (G2m). The reference population were those respondents born in France to two parents born in France. For individuals born in North Africa or born in France to two parents born in North Africa, we took one extra step to better identify North African–origin immigrants and their native-born children as opposed to repatriates and their native-born children. Those reporting that at least one parent spoke to them in Arabic or Berber, alone or in combination with other languages, when they were age 5, or those reporting that their parents spoke to them exclusively in French but were either foreign nationals or naturalized French nationals at the time of the 1999 survey, were identified as North African–origin G1 or G2 immigrants. Individuals who were born in North Africa or born in France to two parents born in North Africa who did not meet these criteria were considered G1 or G2 repatriates and were removed from the analysis. This approach is based on the observation that the vast majority (about 80%) of repatriates were of European descent (Moumen 2010) and were unlikely to have had parents that spoke to them in Arabic or Berber when they were age 5. France-born children of repatriates were thus also unlikely to have had parents that spoke to them in Arabic or Berber when they were age 5. Conversely, immigrants from North Africa and their France-born children were unlikely to have had parents that spoke to them only in French when they were age 5 (Condon and Régnard 2016). Our use of nationality information is based on the fact that repatriates and children of repatriates had by definition a French nationality at birth. The categories ‘foreign’ or ‘French by acquisition’ in the ELM are thus markers of G1 or G2 immigrant status even for those who report that their parents spoke to them only in French. This approach combining language and nationality information is consistent with previous attempts to identify the North African–origin population in France using the EHF data (Tribalat 2004). (See Appendix Section 6 for further details about this approach and a sensitivity analysis.)

In addition to first- and second-generation status, the main sociodemographic characteristic included in this study was educational attainment, measured at baseline in 1999. We used categories following the International Standard Classification of Education (ISCED): ‘primary’ (less than primary and primary); ‘secondary’ (secondary 1^st^ and 2^nd^ cycle); and ‘tertiary’ (post-secondary to pre-university and beyond). Although the ELM included additional socioeconomic background variables, they were all measured at baseline in 1999. Neither retrospective nor prospective measures of socioeconomic status were available. Given these data constraints, we decided to focus on educational attainment as it is the most permanent variable of socioeconomic status and is less subject to reverse causation than variables such as employment status or occupation (Elo 2009).

Our analysis focused on individuals 18 to 64 years old at baseline. The upper age limit was chosen because there were few G2 immigrants above that age in the EHF in 1999, due to the timing of immigration to France from southern Europe and, particularly, North Africa. Substantively, this upper age truncation allowed us to focus on mortality among adults of working ages (18 to 64), a specific age segment associated with the concept of premature mortality.

The overall response rate in the EHF was 79.4%. Among individuals ages 18 to 64 in the EHF, 11.4% had missing information on variables necessary for subgroup attribution (place of birth, parental place of birth, languages, and/or nationality at birth); they were excluded as their first- or second-generation status could not be ascertained. Among those for whom the population subgroup was known, 18.9% could not be matched with the RNIPP and were excluded from the study, as their vital status in 2010 could not be determined. Within the final sample, 2.3% of the individuals had missing information for educational attainment and were assigned to a separate ‘missing’ education category in our models adjusting for educational attainment. (See Appendix Sections 1–3 for more details on missing data and a sensitivity analysis.)

### 2.3 Mortality estimation

We estimated mortality at ages 18 to 64 separately for males and females using a hazard model with age as the duration variable, assuming a Gompertz baseline hazard for the reference population and proportional hazards for our subgroups of interest (G1, G2, and G2m by region of origin). Individuals who reached age 65 prior to the end of the observation period and those whose survival status was ‘alive’ at the end of the observation period were right censored. We converted model parameters into expected probabilities of dying between age 18 and 65 (q_18–65_) for each subgroup of interest using standard life table equations (Preston, Heuveline, and Guillot 2001). We also estimated adjusted hazard ratios for our subgroups of interest, using educational attainment as a covariate.

## 3. Results

Table 1 presents sample sizes at baseline for each subgroup of interest and corresponding deaths occurring prior to age 65 during the observation period. Table 1 also shows how each subgroup is distributed according to background characteristics. Within the age range 18 to 64, G2 subgroups were younger than G1 subgroups for both southern European– and North African-origin individuals. Also, North African–origin subgroups were younger than southern European–origin ones for both G1 and G2, which is expected given that migration flows from North Africa have been more recent than those from southern Europe. G1 southern European–origin males and females tended to be less educated than the reference population. Levels of education for southern European–origin G2 were higher than for their G1 counterparts but still lower than for the reference population. G2 North African–origin males and females had generally lower levels of educational attainment than both their southern European counterparts and the reference population.

**Figure 1:**
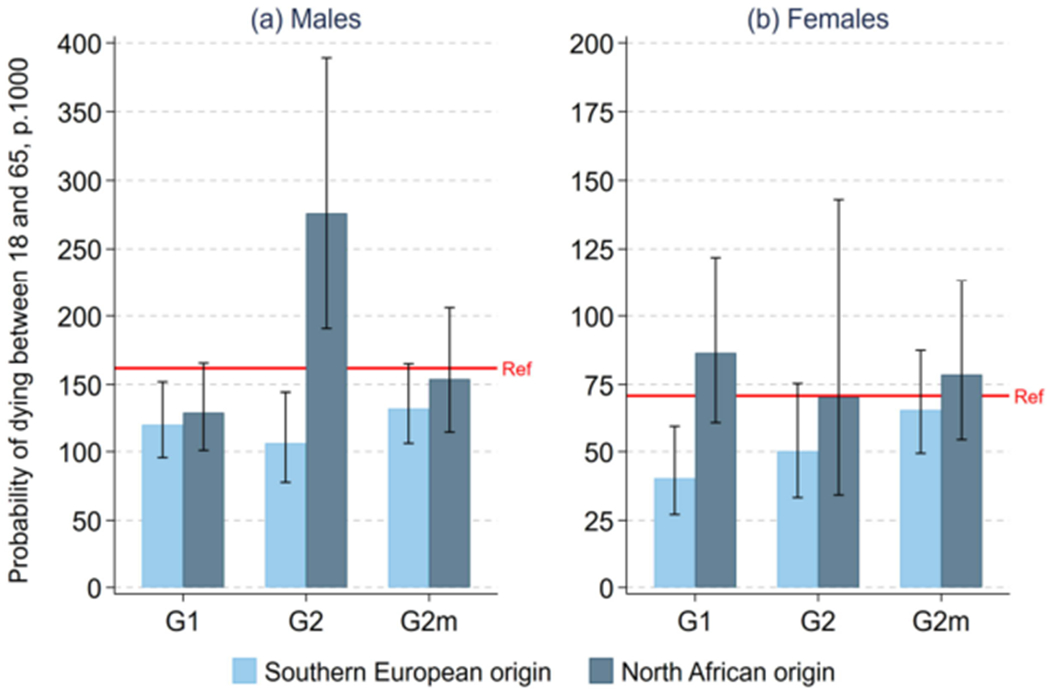
Probability of dying between ages 18 and 65 (q_18–65_) for first- and second-generation immigrant subgroups by region of origin, France, 1999–2010 *Note*: Legend: G1 = first generation; G2 = second generation; G2m = mixed second generation; Ref = reference population (individuals born in France to two parents born in France). *Source:* Echantillon Longitudinal de Mortalité (ELM).

Table 2 shows mortality hazard ratios (HR, with 95% CI) for subgroups of interest, based on our Gompertz hazard model. (See Appendix Tables A-1 and A-2 for an unabridged version of this table.) The unadjusted HRs compare each subgroup with the reference population of individuals born in France to two parents born in France. For ease of interpretation, we show in Figure 1 the corresponding probabilities of dying between age 18 and 65 (q_18-65_), with 95% confidence intervals.

For males (Figure 1, panel a), the estimated level of q_18–65_ for the reference population was 162 per 1,000, a level comparable to results from official vital registration data for a similar period. (See Appendix Section 5 for the details of this comparison.) Results for G1 and G2 subgroups have wide confidence intervals due to small sample sizes. Nonetheless, we observe a strong contrast between generational trajectories for southern European– vs. North African–origin males. For the first generation, we observe low mortality relative to the reference population for both southern European– and North African–origin immigrants, though only marginally significant for North African–origin immigrants. This is consistent with the well-known observation, including in France (Khlat and Courbage 1996; Boulogne et al. 2012), that first-generation immigrants typically experience a mortality advantage. For the second generation, however, a strong contrast appears between southern European– vs. North African–origin individuals. For southern European–origin G2 males, we find a mortality advantage similar to what was observed for their G1 counterparts, with an estimated q_18–65_ level of about 106 per 1,000. For North African–origin G2 males, however, we observe a large amount of excess mortality, with an estimated q_18–65_ level of about 276 per 1,000, which is 1.70 times larger than for the reference population. (As shown in Table 2, this corresponds to a HR of 1.83, with a 95% CI of 1.20 to 2.79.) G2m subgroups have mortality levels closer to the reference population, with differences that are not statistically significant.

Panel b of Figure 1 shows results for females. Confidence intervals are relatively wider than for males, in part because of small numbers of deaths arising from the combination of small sample sizes and lower overall mortality levels for females vs. males. The only subgroup for which we observe a statistically significant difference with the reference population is southern European–origin G1 females, who experience a mortality advantage similar to their male counterparts.

Table 2 also shows how hazard ratios for the different population subgroups change once we adjust for educational attainment. Results for G2 males show that the hazard ratio for those of North African origin decreases somewhat, from 1.83 to 1.68, but remains significant with a 95% confidence interval of 1.10 to 2.56. This suggests that the excess mortality for this subgroup does not simply reflect educational differences. G2 southern European-origin males preserve their mortality advantage once adjusting for education. Results for G2 females remain insignificant after adjusting for education.

## 4. Discussion

Among large EU countries, France stands out as the country with the largest population of second-generation immigrants. Our study documents the existence of a large amount of health disparity by second-generation status in the French context. Specifically, we found a large amount of excess mortality at adult ages among second-generation males of North African origin for the period 1999–2010. This excess mortality is particularly striking for several reasons: it has a large magnitude; it is not present among second-generation males of southern European origin, the other major second-generation subgroup in France, for whom we instead found a mortality advantage; it is not present among North African–origin males of the first generation; and it remains large and significant after taking differences in educational attainment into account. This excess mortality appears to be present only among males; we detect no significant excess among second-generation North African–origin females or indeed in the mixed-second-generation subgroups. To our knowledge, our study is the first one to document these mortality disparities in the French context.

What could explain the excess mortality among second-generation North African–origin males? Differential access to health care is unlikely to be an important explanation, as studies have shown no difference in health care utilization between second-generation immigrants and the reference population in France (Berchet and Jusot 2012). Lack of data on health behaviors and causes of death prevents us from evaluating other proximate determinants, including the role of smoking and alcohol. It is worth noting, however, that the sample of second-generation North African–origin males is quite young, with most individuals at less than 45 years old at the time of the survey. If we top truncate longitudinal follow-up in the sample at age 45, the hazard ratio for this subgroup increases to 2.02 and remains strongly significant (Table A-13). Given that the dominant causes of death among young adult males in a low-mortality country like France are external causes, such as motor vehicle accidents, poisoning, and suicides (Aouba et al. 2011), these causes may be key to understanding the proximate determinants of this excess mortality. The likely importance of external causes of death for this group is corroborated by a study of mortality patterns in Belgium, which found that second-generation North African–origin males had elevated risks of death from drug- and alcohol-related causes (De Grande 2015).

As for distal factors such as socioeconomic status, our model adjusting for education suggests that excess mortality for second-generation North African–origin males is not simply explained by differences in educational attainment. This pattern of excess mortality can perhaps be best understood as part of a broad set of disadvantages for this subgroup in areas including labor market outcomes and income levels (INSEE 2012; Brinbaum, Primon, and Meurs 2016). These disadvantages, which remain after taking background characteristics into account and do not occur for southern European counterparts, have been interpreted as arising in part from discriminatory practices, particularly in the labor market (Silberman and Fournier 1999; Brinbaum, Primon, and Meurs 2016). Our finding of excess mortality among second-generation North African-origin males is consistent with these conditions and raises concerns about their public health consequences in the French context. It is notable that such excess mortality is not found among North African–origin males of the first generation, even though they also experience strong socioeconomic disadvantages (Meurs, Pailhé, and Simon 2006; Brinbaum, Primon, and Meurs 2016). This paradox is well known in the literature and is most convincingly explained by the fact that for first-generation immigrants, the effect of socioeconomic disadvantage on mortality is counteracted by strong migration selection forces – the “healthy migrant effect” – acting in the other direction (Razum et al. 1998; Palloni and Morenoff 2001; Bourbeau 2002; Khlat and Darmon 2003; Crimmins et al. 2005; Gushulak 2007; Riosmena, Wong, and Palloni 2013; Vang et al. 2017). Another factor which may explain the first- vs. second-generation contrast is the fact that first-generation immigrants may retain their country of origin as a frame of reference and from that standpoint may assess their labor market outcomes more favorably than second-generation immigrants for whom the frame of reference is the host country (Heath and Li 2008). Moreover, first-generation immigrants may be more likely to accept labor market disadvantages as part of the “cost” of immigration (Anson 2004). Second-generation immigrants, by contrast, did not decide to immigrate and are likely to be less accepting of such labor market disadvantages. These psychosocial differences may generate poorer health outcomes for second-generation immigrants (Lynch et al. 2000). In the French context, studies have shown that the perception of labor market discrimination is indeed more prevalent among second-generation than first-generation immigrants of the same origin (Meurs, Lhommeau, and Okba 2016), which may translate into worse psychosocial functioning and health outcomes (Paradies 2006; Cobbinah and Lewis 2018).

Although we stress here the pattern of excess mortality among second-generation males of North African origin, we also take note of the mortality advantage we found among those of southern European origin. This new result is surprising and somewhat paradoxical given that this subgroup does not appear to be particularly favored in terms of socioeconomic factors, including education (Table 1), relative to the reference population (Beauchemin, Hamel, and Simon 2016). One possible explanation is the role of social networks. Studies have shown that, for example, second-generation immigrants of Portuguese origin have better labor market outcomes than would be expected on the basis of their educational attainment (Brinbaum, Primon, and Meurs 2016). These labor market advantages have been explained by the role of active social networks facilitating access to jobs (Lebeaux and Degenne 1991; Marry, Fournier-Mearelli, and Kieffer 1995) and could generate better health outcomes. The positive role of social support has also been raised to explain favorable labor and health outcomes among the children of Spanish and Italian immigrants in Switzerland (Bolzman, Fibbi, and Vial 2003; Zufferey 2016). Overall, our results highlight the importance of second-generation status as a significant and previously unknown source of health disparities in France.

This study has some limitations. First, our sample sizes are relatively small. The study’s most important result – a statistically significant (p<.01) excess mortality among second-generation North African–origin adult males – is based on only 22 deaths in the ELM. Even though this result is consistent with other European studies (Scott and Timæus 2013; De Grande et al. 2014; Manhica et al. 2015; Vandenheede et al. 2015; Wallace 2016; Jervelund et al. 2017), our study calls for replication in the French context. However, we are not aware of any alternative source of mortality data in France with variables allowing the proper identification of second-generation immigrants. This current lack of alternative sources makes our results all the more significant, but it also highlights the need for new data collection efforts in France in this area of research. Second, a sizeable proportion (18.9%) of individuals in the sample could not have their vital status ascertained and were thus excluded from the study. To understand the impact of this exclusion on our results, we estimated the effect of background characteristics on the probability of being unmatched using logistic regression. We found that the probability of being unmatched was strongly associated with characteristics indicating lower socioeconomic status, including lower educational attainment and lower occupational status (Table A-8). These associations, combined with the fact that the proportions unmatched were higher among second-generation North African-origin males (24.5%) than among males in the reference population (9.1%), suggest that the high hazard ratios for the former group may in fact be conservative. (See Appendix Section 3 for more details and additional evidence.) Finally, our study lacks proper censoring of individuals who left France permanently during the follow-up period, producing a downward bias in mortality rates. This lack of censoring, however, cannot explain our finding that second-generation North African–origin males experience excess mortality because studies have shown that second-generation immigrants are somewhat more likely to out-migrate – and thus more likely to be affected by a downward bias in mortality estimates – than the reference population with no immigration background (Richard 2004). If anything, this lack of censoring generates conservative estimates of the true amount of excess mortality for this group. (See Appendix Section 4 for an illustration of this mechanism using simulations.)

Despite these limitations, our study provides the first estimates of adult mortality for second-generation immigrants in France. In its 2017 country-specific recommendations for France, the Council of the European Union pointed out that second-generation immigrants in France “face adverse employment outcomes that are not explained by differences in age, education and skills” and that they were only partially closing gaps in educational outcomes. The council recommended “action against discriminatory practices affecting the hiring of non-EU born and second-generation immigrants” (European Commission 2017). Our results for mortality show that the adverse outcomes experienced by second-generation North African-origin males in France also have an important, previously unknown public health dimension. Similar mortality patterns have been found among second-generation males of North African or Middle Eastern origin in other European countries, including Belgium and Sweden (Manhica et al. 2015; Vandenheede et al. 2015). The results for France presented here add to this literature and are particularly significant given the size of the North African–origin population in France and current concerns about the specific conditions they face, including socioeconomic disadvantage and discrimination.

Additional research is urgently needed to further document and understand the causes of these alarming mortality patterns, including the collection of larger mortality samples with variables allowing proper identification of second-generation immigrants together with information on their socioeconomic conditions, health behaviors, morbidity outcomes, and causes of death.

## Supplementary Material

1

## Figures and Tables

**Table 1:** Baseline characteristics of first- and second-generation immigrant subgroups by region of origin in the Echantillon Longitudinal de Mortalité (ELM)

Males	Ref	Southern European origin			North African origin		
		G1	G2	G2m	G1	G2	G2m
**Population**							
N 18–64	74,096	1,788	1,715	2,144	1,640	763	1,810
Deaths 18–64	2,897	64	36	69	55	22	38
**Age (%)**							
18–24	12.9	2.5	15.9	14.2	8.5	43.8	29.2
25–34	23.7	14.3	31.1	23.7	22.7	36.2	41.3
35–44	24.8	26.2	23.0	23.0	25.2	16.5	17.2
45–54	23.2	27.9	16.9	24.2	23.4	2.6	9.0
55–64	15.4	29.1	13.2	14.9	20.2	0.9	3.3
**ISCED education level**							
**18–34 (%)**							
Primary	10.7	36.5	14.8	12.1	23.1	23.2	11.3
Secondary	63.8	51.4	67.6	70.2	54.2	64.1	58.3
Tertiary	25.6	12.1	17.6	17.7	22.7	12.7	30.4
**35–44 (%)**							
Primary	16.3	39.3	20.6	21.7	25.4	25.2	15.9
Secondary	63.9	51.0	63.5	60.3	41.6	52.9	60.9
Tertiary	19.9	9.7	15.9	18.0	33.0	21.9	23.2
**45–64 (%)**							
Primary	29.6	68.3	33.1	32.9	62.0	36.0	22.7
Secondary	53.3	27.2	54.1	54.4	27.8	52.0	54.6
Tertiary	17.1	4.5	12.9	12.8	10.2	12.0	22.7
**Population**							
N 18–64	101,620	2,094	2,408	3,057	1,594	1,045	2,635
Deaths 18–64	1,630	24	22	45	30	7	27
**Age (%)**							
18–24	14.1	3.0	16.1	15.8	14.6	45.6	32.2
25–34	25.2	15.4	35.2	24.8	25.2	38.7	40.6
35–44	24.7	28.6	22.6	24.8	29.1	13.4	17.3
45–54	21.4	28.2	13.3	21.2	19.2	2.1	7.4
55–64	14.6	24.8	12.9	13.4	12.0	0.3	2.5
**ISCED education level**							
**18–34 (%)**							
Primary	9.4	26.5	12.2	11.6	26.8	16.5	11.6
Secondary	58.2	52.3	63.3	59.6	52.3	65.4	59.6
Tertiary	32.4	21.2	24.5	28.8	20.9	18.1	28.8
**35–44 (%)**							
Primary	17.7	44.8	18.8	19.4	48.4	21.4	14.2
Secondary	58.6	44.8	65.1	52.6	35.6	61.8	56.4
Tertiary	23.7	10.4	16.1	28.1	16.0	16.8	29.4
**45–64 (%)**							
Primary	40.0	76.3	47.3	37.5	67.8	54.6	28.6
Secondary	46.2	19.4	46.3	50.3	23.2	31.8	49.6
Tertiary	13.9	4.4	6.5	12.1	9.0	13.6	21.8

*Note*: Ref = individuals born in France to two parents born in France. G1 = first generation. G2 = second generation. G2m = mixed second generation. The education level distributions show percentages among individuals with a non-missing education.

**Table 2:** Mortality hazard ratios (ages 18–64) for first- and second-generation immigrant subgroups by region of origin, France, 1999–2010

	Males				Females			
	Unadjusted HR^1^ (95% CI)		Adjusted HR^2^ (95% CI)		Unadjusted HR^1^ (95% CI)		Adjusted HR^2^ (95% CI)	
Reference population	1		1		1		1	
G1 southern European origin	0.73*	(0.57–0.93)	0.61**	(0.47–0.78)	0.56**	(0.37–0.84)	0.49**	(0.33–0.73)
G1 North African origin	0.79	(0.60–1.03)	0.69**	(0.53–0.90)	1.23	(0.85–1.76)	1.08	(0.75–1.56)
G2 southern European origin	0.64**	(0.46–0.88)	0.62**	(0.44–0.86)	0.70	(0.46–1.06)	0.67	(0.44–1.02)
G2 North African origin	1.83**	(1.20–2.79)	1.68*	(1.10–2.56)	0.99	(0.47–2.09)	0.93	(0.44–1.95)
G2m southern European origin	0.81	(0.63–1.02)	0.78*	(0.61–0.99)	0.92	(0.69–1.24)	0.91	(0.68–1.23)
G2m North African origin	0.95	(0.69–1.31)	0.98	(0.71–1.35)	1.11	(0.76–1.63)	1.14	(0.78–1.67)

*Note*: HR = hazard ratio. CI = confidence interval.

* p<0.05.

** p<0.01.

1 without adjustment for educational attainment.

2 with adjustment for educational attainment, using ISCED categories.

Reference population = individuals born in France to two parents born in France. G1 = first generation. G2 = second generation. G2m = mixed second generation. Models are estimated including residual G1/G2/G2m ‘other regions of origin’ categories. See Table A-1 for an unabridged version of the models’ results.

*Source*: Echantillon Longitudinal de Mortalité (ELM).
