# Supplementary material for: Adult mortality among second-generation immigrants in France: Results from a nationally representative record linkage study
Source: Demogr Res. Author manuscript; Available in PMC 2021 May 12. (PMC8114944; doi:10.4054/demres.2019.40.54)
Supplement: 1 [file NIHMS1695456-supplement-1.pdf]

Appendix

This appendix presents supplementary information for assessing the robustness of the paper's results to various data quality issues (Sections 1–5) and model specifications (Sections 6–7).

The overall conclusion of this sensitivity analysis is that despite various data quality issues inherent to the *Echantillon Longitudinal de Mortalité* (ELM), this data set appears as a reliable source for mortality estimation (Section 5). Moreover, patterns of case exclusion resulting from missing data and other issues suggest that the paper's main result is conservative, that is, that the excess mortality we find among second-generation (G2) immigrant males of North African origin likely underestimates the true amount of excess mortality for this group (Sections 3–4).

1. Response rate in the EHF

The *Etude de l'Histoire Familiale* (EHF) was conducted at the same time as the 1999 census with an average sampling rate of 1/170 for males and 1/110 for females. (Females were oversampled as they were the focus of many of the planned analyses in the EHF.) Eligible individuals were provided with the EHF questionnaire along with the usual census questionnaire. The overall response rate in the EHF (i.e., the proportion of EHF-eligible individuals who completed the census form and also answered the EHF questionnaire) was 79.4%, a response rate that is on par with other large sample surveys commonly used in this literature. An analysis of the probability of response using census variables as explanatory variables shows a lower response among individuals ages 85 and older, individuals who were unmarried, individuals born abroad, or individuals who did not report their level of education in the census (Lefèvre and Filhon 2005).

In order to address these nonresponses, post-stratification weights were provided in the EHF data set, based on the following seven variables: sex, age, education, country of birth, date of arrival in France, region of residence, and size of the place of residence. The results of our paper are based on the unweighted data, because as we show below, our final sample differs from the EHF sample since it excludes individuals for whom survival status is unknown (see Section 3 in this appendix). Nonetheless, an analysis comparing hazard ratios in unweighted vs. weighted models (Tables A-1 and A-2) shows that results for G2 subgroups are robust to the use of post-stratification weights. This suggests that nonresponses in the EHF are unlikely to be the main explanation for the paper's results.

Table A-1: Mortality hazard ratios (ages 18–64) for first- and second-generation immigrant subgroups by region of origin, France, males, 1999–2010; unweighted vs. weighted sample

Males	Model 1 *(baseline)*								Model 2 *(+ education level)*							
	Unweighted				Weighted				Unweighted				Weighted			
	Haz. ratio	SE	Sig	95% CIs	Haz. ratio	SE	Sig	95% CIs	Haz. ratio	SE	Sig	95% CIs	Haz. ratio	SE	Sig	95% CIs
**Generation by region**																
Reference	1				1				1				1			
G1																
North Africa	0.79	0.11	†	*0.60 - 1.03*	0.84	0.13		*0.62 - 1.14*	0.69	0.09	**	*0.53 - 0.90*	0.74	0.11	*	*0.54 - 1.00*
Southern Europe	0.73	0.09	*	*0.57 - 0.93*	0.82	0.13		*0.60 - 1.12*	0.61	0.08	**	*0.47 - 0.78*	0.67	0.11	**	*0.49 - 0.82*
Other regions	0.80	0.09	†	*0.64 - 1.00*	0.83	0.11		*0.63 - 1.09*	0.84	0.10		*0.67 - 1.06*	0.88	0.12		*0.67 - 1.15*
G2																
North Africa	1.83	0.39	**	*1.20 - 2.79*	1.75	0.43	*	*1.09 - 2.82*	1.68	0.36	*	*1.10 - 2.56*	1.60	0.36	†	*1.00 - 2.57*
Southern Europe	0.64	0.11	**	*0.46 - 0.88*	0.65	0.13	*	*0.43 - 0.97*	0.62	0.10	**	*0.44 - 0.86*	0.63	0.10	*	*0.42 - 0.84*
Other regions	1.05	0.16		*0.76 - 1.42*	1.08	0.19		*0.76 - 1.52*	1.04	0.16		*0.77 - 1.40*	1.07	0.16		*0.76 - 1.52*
G2 mixed																
North Africa	0.95	0.16		*0.69 - 1.31*	0.86	0.17		*0.58 - 1.28*	0.98	0.16		*0.71 - 1.35*	0.90	0.18		*0.60 - 1.33*
Southern Europe	0.81	0.10	†	*0.63 - 1.02*	0.97	0.14		*0.73 - 1.28*	0.78	0.10	*	*0.61 - 0.99*	0.94	0.14		*0.71 - 1.24*
Other regions	1.01	0.10		*0.83 - 1.22*	1.13	0.13		*0.91 - 1.42*	1.04	0.10		*0.87 - 1.26*	1.19	0.14		*0.85 - 1.49*
**ISCED education level**																
Tertiary									1				1			
Secondary					*(unadjusted)*				1.71	0.10	**	*1.53 - 1.91*	1.79	0.12	**	*1.57 - 2.04*
Primary									2.41	0.15	**	*2.14 - 2.71*	2.62	0.19	**	*2.28 - 3.02*
Missing									2.46	0.26	**	*2.00 - 3.01*	2.31	0.29	**	*1.81 - 2.96*

Notes: (1) 'reference' refers to individuals born in metropolitan France to two parents born in metropolitan France; (2) significance levels at ** p < 0.01, * p < 0.05, and † p< 0.10.

Table A-2: Mortality hazard ratios (ages 18–64) for first- and second-generation immigrant subgroups by region of origin, France, females, 1999–2010; unweighted vs. weighted sample

	Model 1 *(baseline)*								Model 2 *(+ education level)*							
**Females**	**Unweighted**				**Weighted**				**Unweighted**				**Weighted**			
	**Haz. ratio**	**SE**	**Sig**	**95% CIs**	**Haz. ratio**	**SE**	**Sig**	**95% CIs**	**Haz. ratio**	**SE**	**Sig**	**95% CIs**	**Haz. ratio**	**SE**	**Sig**	**95% CIs**
**Generation by region**																
Reference	1				1				1				1			
G1																
North Africa	1.23	0.23		*0.85 - 1.76*	1.29	0.27		*0.85 - 1.95*	1.08	0.20		*0.75 - 1.56*	1.15	0.25		*0.75 - 1.75*
Southern Europe	0.56	0.11	**	*0.37 - 0.84*	0.52	0.13	**	*0.33 - 0.84*	0.49	0.10	**	*0.33 - 0.73*	0.46	0.11	**	*0.29 - 0.74*
Other regions	1.05	0.14		*0.81 - 1.36*	1.00	0.15		*0.74 - 1.35*	1.09	0.15		*0.84 - 1.41*	1.02	0.16		*0.75 - 1.39*
G2																
North Africa	0.99	0.38		*0.47 - 2.09*	0.93	0.40		*0.40 - 2.16*	0.93	0.35		*0.44 - 1.95*	0.87	0.37		*0.37 - 2.02*
Southern Europe	0.70	0.15	†	*0.46 - 1.06*	0.67	0.16		*0.42 - 1.08*	0.67	0.15	†	*0.44 - 1.02*	0.65	0.15	†	*0.41 - 1.05*
Other regions	0.86	0.19		*0.56 - 1.33*	1.04	0.26		*0.64 - 1.70*	0.85	0.19		*0.55 - 1.31*	1.03	0.25		*0.63 - 1.67*
G2 mixed																
North Africa	1.11	0.22		*0.76 - 1.63*	0.89	0.19		*0.58 - 1.37*	1.14	0.22		*0.78 - 1.67*	0.92	0.20		*0.60 - 1.40*
Southern Europe	0.92	0.14		*0.69 - 1.24*	1.07	0.19		*0.76 - 1.51*	0.91	0.14		*0.68 - 1.23*	1.06	0.18		*0.76 - 1.49*
Other regions	1.33	0.15	*	*1.06 - 1.67*	1.53	0.21	**	*1.18 - 1.99*	1.34	0.15	**	*1.07 - 1.68*	1.55	0.21	**	*1.19 - 2.02*
**ISCED education level**																
Tertiary									1				1			
Secondary					*(unadjusted)*				1.71	0.10	**	*1.53 - 1.91*	1.79	0.12	**	*1.57 - 2.04*
Primary									2.41	0.15	**	*2.14 - 2.71*	2.62	0.19	**	*2.28 - 3.02*
Missing									2.46	0.26	**	*2.00 - 3.01*	2.31	0.29	**	*1.81 - 2.96*

Note: (1) 'reference' refers to individuals born in metropolitan France to two parents born in metropolitan France; (2) significance levels at ** p < 0.01, * p < 0.05, and † p< 0.10.

2. Missing data in the EHF sample

Our results exclude EHF individuals who could not be allocated to a specific population group due to missing values on the relevant variables (place of birth, parental place of birth, languages, and nationality at birth). Figures A-1 and A-2 show the process of subgroup attribution and the extent of missing data at each step. In these figures, the missing cases in a given box correspond to individuals who had missing values on variables needed for the attribution of categories at the same level.

Among males, a total of 14,405 individuals, or 12.1% of the total EHF sample of 119,473 individuals, had missing information on variables necessary for subgroup attribution. In terms of proportion missing among non-missing individuals in the previous level, the largest percentage is for individuals born in France who didn't report the necessary information for allocation in a specific reference or G2 category (9.6%).

The amount of missing data was slightly lower for females. Out of a total of 183,814 females in the EHF, 20,064 (10.9%) had missing information for subgroup attribution. Among females born in France, 8.3% could not be attributed to a specific reference or G2 category.

Figure A-1: Flow chart representing how individuals in the EHF are attributed to different population subgroups, males

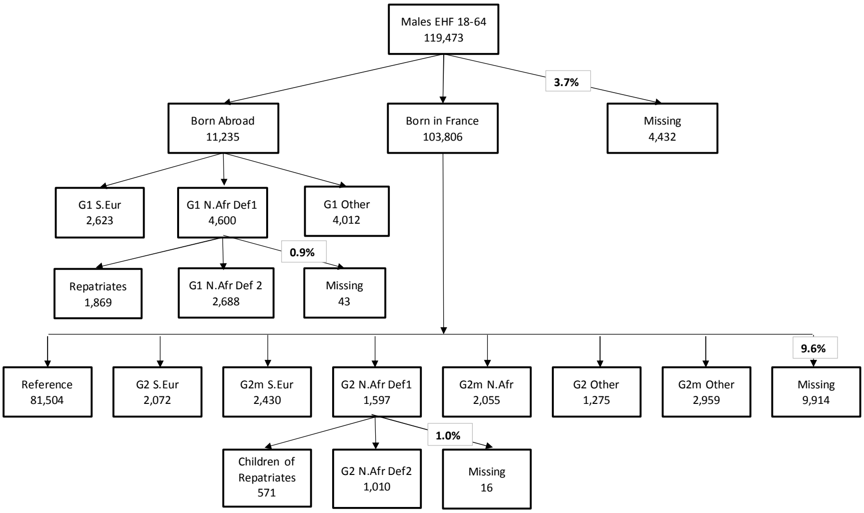

Note: G1 = first generation; G2 = second generation; G2m = mixed second generation. N.Afr = North African origin: S.Eur = southern European origin. G1/G2 N.Afr Def 1: first- or second-generation immigrants of North African origin, based on country of birth information only. G1/G2 N.Afr Def 2: first- or second-generation immigrants of North African origin, based on country of birth, language, and nationality information (see text for details).

Figure A-2: Flow chart representing how individuals in the EHF are attributed to different population subgroups, females

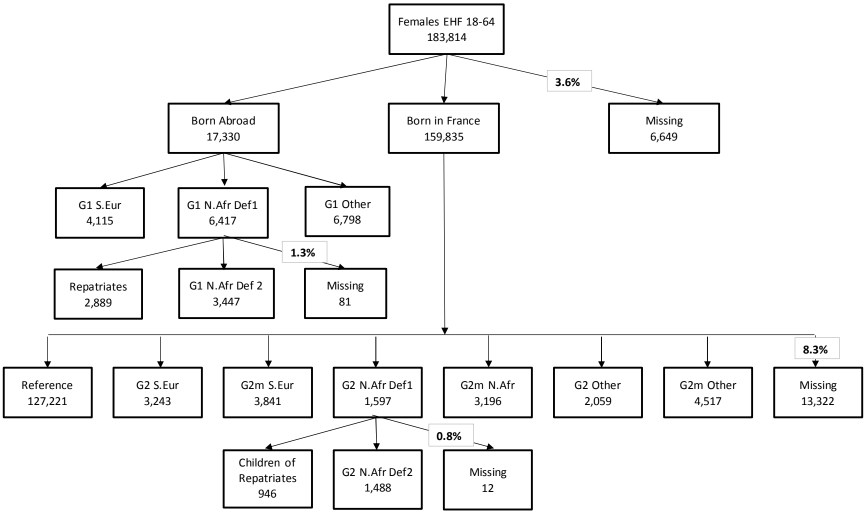

Note: See Figure A-1.

Table A-3 shows how these native-born individuals with missing information for G2 vs. reference subgroup attribution were distributed according to the type of missing information (paternal and/or maternal place of birth).

Table A-3: Distribution of native-born individuals in the EHF with missing paternal and/or maternal country of birth

Type of missing parental place of birth	Males	Females
Both parents missing	5,951	7,302
One parent France, other parent missing	3,663	5,516
One parent southern Europe, other parent missing	98	165
One parent North Africa, other parent missing	92	136
One parent other countries, other parent missing	110	203
Total	9,914	13,322

While the majority of these missing cases had missing place of birth for both parents, a large proportion of these missing cases declared one parent born in France (36.9% for males and 41.4% for females). While we cannot attribute these individuals to a specific subgroup category, we do know that they do not belong to the two main G2 categories of interest in the paper (G2 southern Europe and G2 North Africa).

However, they may or may not belong to the reference category (born in France to two parents born in France).

In order to examine the robustness of our results to this specific type of missing information (one parent France, other parent missing), we estimated our main model using two extreme scenarios: (1) a scenario in which none of these individuals belong to the reference category; (2) a scenario in which all of these individuals belong the reference category.

The first scenario is examined in a model treating native-born individuals with one parent born in France and the other parent with missing country of birth (one parent France, one unknown) as a separate category. Results from this model are shown in Table A-4. The second scenario is examined in a version of the model that includes all these individuals in the reference category. Results are shown in Table A-5.

Results show that while these G2 missing cases have higher mortality than the reference category (Table A-4), the hazard ratios for G2 subgroups of interest are robust to these different model specifications (Tables A-4 and A-5). In particular, the excess mortality among G2 North African males and the mortality advantage among G2 southern European males discussed in the paper are not affected by the choice of scenario for handling these G2 missing cases. While this robustness test does not address all the G2 missing cases, it addresses a substantial portion of them. The remaining cases with missing parental place of birth not addressed by this robustness test are 6,251 for males (6.0% of all native-born males) and 7,806 for females (4.9% of all native-born females).

Table A-4: Mortality hazard ratios (ages 18–64) for first- and second-generation immigrant subgroups by region of origin, France, 1999–2010; native-born individuals with 'one parent France, one unknown' (G2 missing) treated as separate category

	Model 1 *(baseline)*						Model 2 *(+ education level)*					
	Haz. ratio	SE	Sig	95% CIs			Haz. ratio	SE	Sig	95% CIs		
**Males**												
**Generation by region**												
Reference	1						1					
G1												
North Africa	0.79	0.11	†	*0.60*	–	*1.03*	0.69	0.09	**	*0.53*	–	*0.90*
Southern Europe	0.73	0.10	*	*0.57*	–	*0.93*	0.61	0.08	**	*0.48*	–	*0.78*
Other regions	0.80	0.10	†	*0.64*	–	*1.00*	0.84	0.10		*0.67*	–	*1.06*
G2												
North Africa	1.82	0.39	**	*1.19*	–	*2.77*	1.67	0.36	**	*1.09*	–	*2.55*
Southern Europe	0.64	0.11	**	*0.46*	–	*0.88*	0.61	0.10	**	*0.44*	–	*0.85*
Other regions	1.05	0.16		*0.78*	–	*1.42*	1.04	0.16		*0.77*	–	*1.41*

Table A-4: (Continued)

	Model 1 *(baseline)*						Model 2 *(+ education level)*					
	Haz. ratio	SE	Sig	95% CIs			Haz. ratio	SE	Sig	95% CIs		
**Males**												
G2 mixed												
North Africa	0.94	0.15		*0.69*	–	*1.30*	0.98	0.16		*0.71*	–	*1.35*
Southern Europe	0.81	0.10	†	*0.64*	–	*1.02*	0.78	0.09	*	*0.61*	–	*0.99*
Other regions	1.01	0.10		*0.83*	–	*1.22*	1.05	0.10		*0.87*	–	*1.26*
G2 missing												
One parent France, one unknown	1.24	0.11	**	*1.05*	–	*1.46*	1.15	0.10	†	*0.98*	–	*1.37*
**ISCED education level**												
Tertiary							1					
Secondary			*(unadjusted)*				1.74	0.10	**	*1.56*	–	*1.93*
Primary							2.47	0.15	**	*2.20*	–	*2.77*
Missing							2.44	0.25	**	*2.00*	–	*2.97*
**Females**												
**Generation by region**												
Reference	1						1					
G1												
North Africa	1.23	0.23		*0.85*	–	*1.76*	1.09	0.20		*0.76*	–	*1.56*
Southern Europe	0.56	0.12	**	*0.37*	–	*0.84*	0.49	0.10	**	*0.33*	–	*0.74*
Other regions	1.05	0.14		*0.81*	–	*1.36*	1.09	0.14		*0.84*	–	*1.41*
G2												
North Africa	0.99	0.38		*0.47*	–	*2.08*	0.93	0.35		*0.44*	–	*1.95*
Southern Europe	0.70	0.15	†	*0.46*	–	*1.06*	0.67	0.14	†	*0.44*	–	*1.03*
Other regions	0.86	0.19		*0.56*	–	*1.33*	0.85	0.19		*0.55*	–	*1.31*
G2 mixed												
North Africa	1.11	0.22		*0.76*	–	*1.62*	1.14	0.22		*0.78*	–	*1.67*
Southern Europe	0.91	0.14		*0.69*	–	*1.24*	0.92	0.14		*0.68*	–	*1.23*
Other regions	1.33	0.15	**	*1.06*	–	*1.67*	1.34	0.15	**	*1.07*	–	*1.68*
G2 missing												
One parent France, one unknown	1.45	0.16	**	*1.17*	–	*1.79*	1.37	0.15	**	*1.11*	–	*1.70*
**ISCED education level**												
Tertiary							1					
Secondary			*(unadjusted)*				1.44	0.10	**	*1.26*	–	*1.65*
Primary							1.82	0.13	**	*1.57*	–	*2.10*
Missing							2.08	0.26	**	*1.63*	–	*2.65*

Note: (1) 'reference' refers to individuals born in metropolitan France to two parents born in metropolitan France; (2) significance levels at ** p < 0.01, * p < 0.05, and † p< 0.10.

Table A-5: Mortality hazard ratios (ages 18–64) for first- and second-generation immigrant subgroups by region of origin, France, 1999–2010; native-born individuals with 'one parent France, one unknown' included in the reference category

	**Model 1** *(baseline)*				**Model 2** *(+ education level)*			
**Males**	**Haz. ratio**	**SE**	**Sig**	**95% CIs**	**Haz. ratio**	**SE**	**Sig**	**95% CIs**
**Generation by region**								
Reference	1				1			
G1								
North Africa	0.78	0.11	†	0.60 – 1.02	0.68	0.09	**	0.52 – 0.89
Southern Europe	0.72	0.10	*	0.56 – 0.93	0.60	0.08	**	0.47 – 0.77
Other regions	0.79	0.10	*	0.63 – 0.99	0.84	0.10		0.67 – 1.05
G2								
North Africa	1.80	0.39	**	1.18 – 2.74	1.66	0.36	**	1.09 – 2.53
Southern Europe	0.63	0.11	**	0.45 – 0.87	0.61	0.10	**	0.44 – 0.85
Other regions	1.04	0.16		0.77 – 1.41	1.03	0.16		0.77 – 1.40
G2 mixed								
North Africa	0.94	0.15		0.68 – 1.29	0.97	0.16		0.70 – 1.34
Southern Europe	0.80	0.10	†	0.63 – 1.01	0.77	0.09	*	0.61 – 0.98
Other regions	1.00	0.10		0.83 – 1.21	1.04	0.10		0.86 – 1.25
**ISCED education level**								
Tertiary					1			
Secondary			(unadjusted)		1.74	0.10	**	1.56 – 1.94
Primary					2.48	0.15	**	2.21 – 2.78
Missing					2.45	0.25	**	2.01 – 2.99
**Females**								
**Generation by region**								
Reference	1				1			
G1								
North Africa	1.21	0.22		*0.84 – 1.73*	1.07	0.20		*0.74 – 1.54*
Southern Europe	0.55	0.11	**	*0.37 – 0.82*	0.48	0.10	**	*0.32 – 0.73*
Other regions	1.03	0.14		*0.80 – 1.34*	1.07	0.14		*0.83 – 1.39*
G2								
North Africa	0.97	0.37		*0.46 – 2.05*	0.91	0.35		*0.43 – 1.92*
Southern Europe	0.69	0.15	†	*0.45 – 1.04*	0.66	0.14	†	*0.44 – 1.01*
Other regions	0.85	0.19		*0.55 – 1.31*	0.84	0.18		*0.55 – 1.29*
G2 mixed								
North Africa	1.09	0.21		*0.74 – 1.60*	1.12	0.22		*0.77 – 1.64*
Southern Europe	0.91	0.14		*0.68 – 1.22*	0.91	0.14		*0.67 – 1.21*
Other regions	1.31	0.15	**	*1.05 – 1.64*	1.32	0.15	**	*1.06 – 1.66*
**ISCED education level**								
Tertiary					1			
Secondary			*(unadjusted)*		1.45	0.10	**	*1.27 – 1.65*
Primary					1.83	0.13	**	*1.59 – 2.11*
Missing					2.11	0.26	**	*1.65 – 2.69*

Note: (1) 'reference' refers to individuals born in metropolitan France to two parents born in metropolitan France; (2) significance levels at ** p < 0.01, * p < 0.05, and † p< 0.10.

3. Missing survival status among EHF individuals

The results presented in the paper are based on the ELM data set, which includes only those EHF individuals who could be matched with the RNIPP (National Directory for the Identification of Natural Persons), as explained in the paper. Individuals who could not be matched with the RNIPP had an unknown survival status and were thus excluded from the final sample. As explained in the paper, the matching procedure was based on information on first and last names as well as date of birth.

The overall proportion of the EHF individuals who could be matched with the RNIPP is 87.3% for males and 76.3% for females. Tables A-6 and A-7 show proportions matched for each of the subgroups identified in the above flowcharts. Results for males show that the proportions matched were highest for the reference population (90.9%) and lowest for the foreign-born groups (68.2% for G1 southern Europe and 61.0% for G1 North Africa). Second-generation immigrant groups were somewhere in between, with 82.8% matched for G2 southern Europe and 75.5% for G2 North Africa. Repatriates, whether G1 or G2, had proportions matched that were close to the reference population, which is consistent with the expectation that a large majority of repatriates had French last names (vs. Arabic last names for the North African immigrants) that were likely more easily matched with the RNIPP.

Proportions matched among females were generally lower than for males, presumably because of changes in last names after marriage, making it more difficult to match female respondents with the RNIPP.

Table A-6: Counts and proportions of EHF individuals matched with the RNIPP, by population subgroup; males

**Males, EHF, 18–65**					
**Generation by region of origin**			**Total**	**Matched**	
				n	**%**
Foreign-born	G1 southern Europe		2,623	1,788	68.2
	G1 North Africa (Definition 1)	Definition 2	2,688	1,640	61.0
		Repatriates	1,869	1,637	87.6
		Missing	43	36	83.7
	G1 other		4,012	2,419	60.3
Native-born	Reference population		81,504	74,096	90.9
	G2 southern Europe		2,072	1,715	82.8
	G2 mixed southern Europe		2,430	2,144	88.2
	G2 North Africa (Definition 1)	Definition 2	1,010	763	75.5
		Repatriates	571	485	84.9
		Missing	16	12	75.0
	G2 mixed North Africa		2,055	1,810	88.1
	G2 other		1,275	1,017	79.8
	G2 mixed other		2,959	2,619	88.5
	One parent born in France, other parent missing		3,641	3,194	87.7
	Missing		6,273	5,210	83.1
Missing			4,432	3,722	84.0
Total			119,473	104,307	87.3

Note: G1 = first generation; G2 = second generation; G2 mixed = mixed second generation. G1/G2 North Africa Definition 1: first- or second-generation immigrants of North African origin, based on country of birth information only. G1/G2 North Africa Definition 2: first- or second-generation immigrants of North African origin, based on country of birth, language, and nationality information (see text for details).

Table A-7: Counts and proportions of EHF individuals matched with the RNIPP, by population subgroup; females

Females, EHF, 18–65					
Generation by region of origin			Total	Matched *n*	%
	G1 southern Europe		4,115	2,094	50.9
		Definition 2	3,447	1,594	46.2
Foreign-born	G1 North Africa (Definition 1)	Repatriates	2,889	2,110	73.0
		Missing	81	47	58.0
	G1 other		6,798	3,566	52.5
	Reference population		127,211	101,620	79.9
	G2 southern Europe		3,243	2,408	74.3
	G2 mixed southern Europe		3,841	3,057	79.6
		Definition 2	1,488	1,045	70.2
	G2 North Africa (Definition 1)	Repatriates	946	730	77.2
Native-born		Missing	12	10	83.3
	G2 mixed North Africa		3,196	2,635	82.4
	G2 other		2,059	1,487	72.2
	G2 mixed other		4,517	3,547	78.5
	One parent born in France, other parent missing		5,478	4,149	75.7
	Missing		7,844	5,320	67.8
Missing			6,649	4,921	74.0
Total			183,814	140,340	76.3

Note: See Table A-6.

In our analysis, all the individuals who were unmatched with the RNIPP were removed from the analysis as their survival status could not be ascertained. In order to assess the impact of these matching failures on our mortality estimates, we examined which background variables were associated with the probability of being unmatched using multivariate logistic regression. Results (Table A-8) confirm the matching patterns by population subgroup observed in Tables A-6 and A-7. Additionally, they show that individuals with lower education or who were unemployed were more likely to be unmatched. Being married was associated with a higher likelihood of being unmatched for females but not for males, which is consistent with the expectation that changes in last name make matching more problematic. Overall, Table A-8 suggests an overall downward bias in mortality estimates in the ELM due to selective exclusion of individuals from lower SES categories. Given the lower proportions matched among G2 North Africa by comparison with the reference category, the downward bias is likely to be larger for this group, suggesting that the excess mortality we find among G2 North African–origin males underestimates the true amount of excess mortality for this group.

Table A-8: Logistic regression for the probability of being unmatched with the RNIPP, EHF 1999

	Males						Females					
	HR	S.E.	Sig	95% CIs			HR	S.E.	Sig	95% CIs		
**Generation by region of origin**												
Ref	**1**						**1**					
G2												
North Africa	2.97	0.23	**	2.56	–	3.46	2.62	0.16	**	2.34	–	2.96
Southern Europe	1.82	0.12	**	1.61	–	2.06	1.46	0.06	**	1.34	–	1.59
Other	2.03	0.16	**	1.74	–	2.36	1.48	0.08	**	1.33	–	1.64
G2 mixed												
North Africa	1.27	0.09	**	1.10	–	1.46	1.19	0.06	**	1.08	–	1.32
Southern Europe	1.18	0.08	**	1.04	–	1.35	1.05	0.04		0.97	–	1.14
Other	1.17	0.07	**	1.04	–	1.32	1.08	0.04	*	1.00	–	1.16
G1												
North Africa	5.55	0.24	**	5.10	–	6.04	4.01	0.15	**	3.73	–	4.31
Southern Europe	4.10	0.18	**	3.76	–	4.48	3.07	0.10	**	2.88	–	3.28
Other	6.17	0.22	**	5.76	–	6.62	3.56	0.09	**	3.38	–	3.75
**Age**												
18–24	**1**						**1**					
25–29	1.14	0.05	**	1.04	–	1.25	1.17	0.04	**	1.09	–	1.26
30–34	1.18	0.06	**	1.07	–	1.29	1.18	0.04	**	1.10	–	1.26
35–39	1.16	0.06	**	1.06	–	1.28	1.29	0.05	**	1.20	–	1.39
40–44	1.21	0.06	**	1.10	–	1.34	1.47	0.05	**	1.37	–	1.58
45–49	1.29	0.06	**	1.17	–	1.43	1.62	0.06	**	1.52	–	1.75
50–54	1.30	0.07	**	1.18	–	1.44	1.66	0.06	**	1.54	–	1.78
55–59	1.28	0.07	**	1.15	–	1.44	1.57	0.06	**	1.45	–	1.70
60–64	1.22	0.09	**	1.05	–	1.41	1.46	0.06	**	1.34	–	1.59
**ISCED education level**												
Tertiary	**1**						**1**					
Secondary	1.12	0.03	**	1.06	–	1.18	1.09	0.02	**	1.05	–	1.12
Primary	1.40	0.05	**	1.31	–	1.49	1.35	0.03	**	1.30	–	1.41
Missing	2.22	0.11	**	2.01	–	2.45	2.45	0.08	**	2.30	–	2.62
**Marital status**												
Single	**1**						**1**					
Married	0.86	0.05	**	1.31	–	0.90	2.14	0.04	**	2.06	–	2.23
Widowed	1.00	0.03		1.06	–	1.26	2.96	0.11	**	2.75	–	3.18
Divorced	0.95	0.11		2.01	–	1.05	2.14	0.06	**	2.03	–	2.27
**Economic activity**												
Employed	**1**						**1**					
Studying	0.95	0.05		0.86	–	1.06	1.08	0.05	†	1.00	–	1.17
Unemployed	1.06	0.04		0.99	–	1.13	1.07	0.02	**	1.03	–	1.13
Retired	0.89	0.05	*	0.80	–	1.00	1.09	0.03	**	1.03	–	1.16
At home, long-term sick	1.15	0.07	*	1.02	–	1.29	1.06	0.02	**	1.03	–	1.10
Missing	1.64	0.08	**	1.49	–	1.80	1.73	0.05	**	1.63	–	1.83

The conclusion that the ELM produces conservative estimates of the true amount of excess mortality for G2 North African–origin males is further supported by a comparison of education distributions for all individuals (whether matched or unmatched in the RNIPP) vs. the education distributions for matched individuals only (i.e., those on the basis of whom mortality hazard ratios are estimated). Results (Table A-9) show that for the reference population and G2 southern Europe, there is little distortion in educational distribution for the matched sample vs. the entire EHF sample. For the G2 North Africa group, however, the matched sample is substantially distorted toward higher education categories. The proportions with primary education for this group are indeed systematically lower in the matched sample than in the entire EHF sample. This further suggests that the excess mortality we find for second-generation North African–origin males underestimates the true amount of excess mortality for this group.

Table A-9: Distribution (%) of reference and second-generation immigrant subgroups by educational attainment, all EHF individuals vs. EHF individuals matched with the RNIPP

ISCED education level	Reference		G2 southern Europe		G2 North Africa	
	All	Matched	All	Matched	All	Matched
**Males**						
**18–34**						
Primary	11.0	10.7	16.2	14.8	23.6	19.1
Secondary	63.7	63.8	66.5	67.6	64.3	63.2
Tertiary	25.3	25.6	17.3	17.6	12.1	17.7
Total	100.0	100.0	100.0	100.0	100.0	100.0
**35–44**						
Primary	16.5	16.3	21.4	20.6	26.7	18.9
Secondary	63.8	63.9	65.0	63.5	52.0	56.8
Tertiary	19.7	19.9	13.7	15.9	21.3	24.3
Total	100.0	100.0	100.0	100.0	100.0	100.0
**45–64**						
Primary	30.1	29.6	33.8	33.1	52.4	35.7
Secondary	53.2	53.3	53.2	54.1	38.1	50.0
Tertiary	16.8	17.1	13.0	12.9	9.5	14.3
Total	100.0	100.0	100.0	100.0	100.0	100.0
**Females**						
**18–34**						
Primary	10.0	9.4	13.7	12.2	18.6	14.8
Secondary	58.1	58.2	63.7	63.3	64.6	62.3
Tertiary	31.9	32.4	22.7	24.5	16.9	22.9
Total	100.0	100.0	100.0	100.0	100.0	100.0

Table A-9: (Continued)

ISCED education level	Reference		G2 southern Europe		G2 North Africa	
	All	Matched	All	Matched	All	Matched
**Females**						
**35–44**						
Primary	18.8	17.7	19.5	18.8	24.1	17.8
Secondary	58.3	58.6	64.2	65.1	61.6	60.1
Tertiary	23.0	23.7	16.3	16.1	14.3	22.1
Total	100.0	100.0	100.0	100.0	100.0	100.0
**45–64**						
Primary	40.7	40.0	47.0	47.3	63.9	44.4
Secondary	45.5	46.2	45.9	46.3	25.0	42.6
Tertiary	13.8	13.9	7.1	6.5	11.1	13.0
Total	100.0	100.0	100.0	100.0	100.0	100.0

4. Impact of out-migration on mortality estimates

As explained in the text, the ELM does not contain information on international out-migrations. As a result, individuals who leave France during the follow-up period (1999–2010) erroneously remain in the risk pool, producing a downward bias in mortality rates. This is a classic bias inherent to many studies in this literature (Palloni and Arias 2004). In the paper, we explain that G2 individuals are more likely to out-migrate than individuals with no immigration background, implying that the downward bias in mortality rates will be larger for G2 individuals than for the reference population. We conclude that the excess mortality we find among G2 North African males cannot be explained by a lack of information on international out-migrations.

Here we illustrate this conclusion with simulations. These simulations were carried out using a Poisson regression framework with death and exposure terms broken down into two periods (1999–2004 and 2005–2010). We applied various rates of out-migration to our two main G2 groups (North Africa and southern Europe) and examined the impact of these out-migration scenarios on incidence rate ratios. Out-migrations were uniformly distributed during the follow-up period. For example, the scenario with a 10% out-migration rate assumes that by the end of the follow-up period, 10% of the baseline G2 population left France, generating a 2.5% decrease in exposure for the period 1999–2004 and a 7.5% decrease in exposure for the period 2005–2010. The Poisson model is then estimated with dummy variables for population subgroup, age, and time period as explanatory variables. Out-migration rates in these simulations correspond to the amount of additional out-migration that these G2 groups experience relative to the reference population. (If all groups experienced the same rates of out-migration, hazard ratios would remain unbiased.)

Results are shown in Figure A-3. In this figure, the baseline scenario with an out-migration rate of 0% produces results that correspond to those presented in the paper, which is expected given that the paper does not adjust for out-migration. (The use of a Poisson framework here produces almost identical results as the Gompertz framework used in the paper.) When out-migration is introduced, the incidence ratios systematically increase, illustrating the point made in the paper that our hazard ratios underestimate true hazard ratios whenever G2 groups experience more out-migration than the reference population. Results for G2 North African males confirm the paper's conclusion that our lack of information on out-migrations produces conservative estimates of the true hazard ratios for this population. Interestingly, our simulations also show that the mortality advantage we find among G2 southern European males is also unlikely to be explained by out-migration. Even in a scenario in which 15% of individuals at baseline leave by the end of the observation period, incidence rate ratios for this group would still remain below 1 and statistically significant. Results for females show that incidence rate ratios remain insignificant whatever the amount of assumed out-migration.

Figure A-3: Mortality incidence rate ratios (ages 18–64) for second-generation immigrant subgroups estimates using different out-migration scenarios, France, 1999–2010

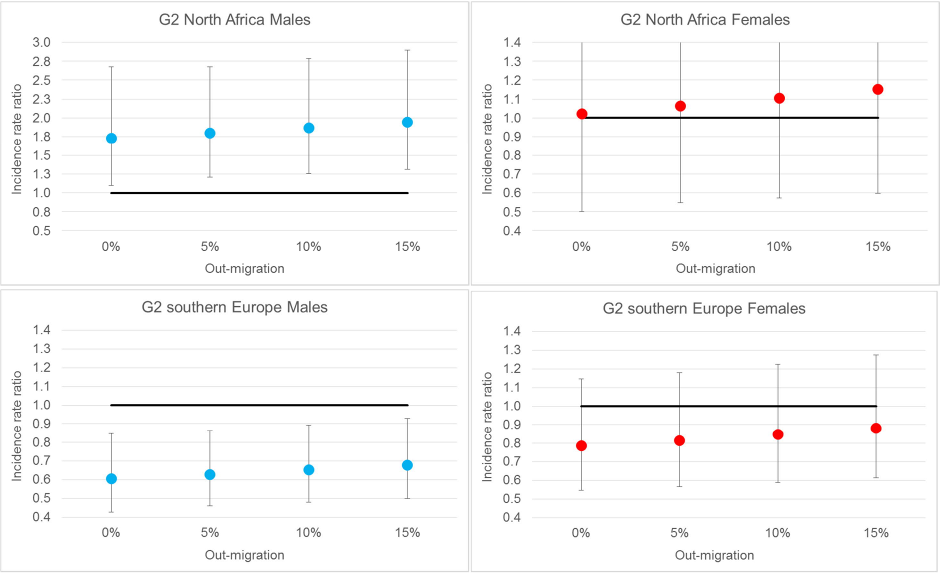

A different type of mechanism that could potentially affect our mortality estimates involves selective out-migration of healthier G2 individuals prior to the baseline year of 1999. Indeed, if such selective out-migration was taking place before 1999, this would make the baseline sample in 1999 less healthy than in the absence of out-migration, potentially generating an upward bias in mortality estimates. The importance of this mechanism is difficult to assess in the absence of longitudinal follow-up since birth. However, it is unlikely that the excess mortality we observe during the follow-up period among G2 North African–origin males would be explained by this mechanism, because out-migration among this population, while higher than for the reference population, is estimated to be rather small (Richard 2004). Also, a recent study has shown that the mortality disadvantage among second-generation North African individuals is already observed at infant ages, a result that cannot be explained by left-truncation bias (Wallace, Guillot, and Khlat 2019).

5. Overall quality of the ELM for mortality estimation purposes

The previous sections examine the robustness of our mortality estimates to various sources of errors in the ELM. In this section, we examine the overall quality of the ELM data for mortality estimation purposes by comparing ELM-based adult mortality estimates with mortality estimates based on official exhaustive census and vital registration (VR) data. This unlinked data forms the basis for the calculation of official life tables in France and thus constitutes a useful comparison point for evaluating the ELM-based mortality data.

This comparison is possible for only the native-born and the foreign-born, since G2 status cannot be derived from the information available on death certificates. We were able to access VR death data by nativity for 2005–2009, a period that is not exactly the same but overlaps with the time frame of the ELM (1999–2010). Exposure terms by sex and nativity for the period 2005–2009 were derived from census information. Deaths and exposure terms were then combined to calculate age-specific mortality rates, which were then converted into probabilities of dying between age 18 and 65 using standard life table methodologies. (For more information about these sources, see Guillot et al. 2018.)

Table A-10 compares these probabilities of dying calculated on the basis of these two different data sources. Results show that despite its limitations, the ELM produces mortality estimates that are reassuringly close to those based on exhaustive official VR data. The ELM has a tendency to underestimate mortality, which is expected given the discussion of biases in the preceding sections. Nonetheless, the difference is never more than 9%. The difference is even smaller for the foreign-born population, despite the

specific data limitations inherent in this population. Overall, this comparison suggests that despite its limitations, the ELM appears as a reliable source of mortality information for these population subgroups in France.

Table A-10: Comparison of ELM- vs. vital registration–based estimates of the probability of dying between age 18 and 65 ($q_{18–65}$), by sex and nativity

		1999-2010<br>ELM	2005-2009<br>VR	Difference
Males	Native-born	0.16067	0.17353	–7.4%
	Foreign-born	0.12738	0.13135	–3.0%
Females	Native-born	0.07137	0.07845	–9.0%
	Foreign-born	0.06516	0.06630	–1.7%

6. Defining the G1 and G2 North African–origin group

According to the French national statistical office, an immigrant is a person residing in France who was born abroad and had a foreign nationality at birth. Building on that definition, a second-generation immigrant is a person residing in France who was born in France and had at least one immigrant parent.

As explained in the text, we generally relied only on country of birth information for determining first- and second-generation immigrant status. The reason is that although we have information on the respondent's country of birth, nationality at birth, and parental country of birth in our data, we do not have information on parental nationality at birth, which would be necessary for determining second-generation immigrant status as officially defined. Using country of birth as the sole piece of information for identifying immigrants is an acceptable approximation for most countries of birth because the proportion of foreign-born individuals who have a French nationality at birth is negligible in most cases. In the case of North African countries, however, this approximation is problematic. Indeed a substantial share of France's North Africa–born population includes 'repatriates,' a group of individuals who were born in Algeria during the colonial period and relocated to France following Algeria's independence in 1962. Repatriates include three main categories: (1) individuals of European descent; (2) North African Jews; (3) some North African Muslims, including soldiers who fought with the French army against independence (also called 'harkis') and officials of the former colonial administration who feared for their security in post-independence Algeria. Typically these 'repatriates' are not considered immigrants in the French context because not only were they French by birth but they did not lose their French nationality after Algeria's independence (Beauchemin, Hamel, and Simon

2016). Available estimates show that among repatriates, individuals of European descent constituted by far the largest category (about 80%) (Moumen 2010).

In response to the specificity of North African countries, and in the absence of information on parental nationality at birth, we used additional variables to identify immigrants (vs. repatriates) from North Africa and their native-born children. As explained in the text, our approach uses language and nationality information for this distinction.

This approach is not perfect. In particular, it tends to classify repatriates of the third category (North African Muslims) as immigrants rather than repatriates. We believe this is not problematic for our paper because (1) North African Muslims represent a small percentage of the 'repatriates' category (about 9%); and (2) although they are not considered 'immigrants' per se due to their nationality status, they share the same ethnic background with their immigrant counterparts and are thus likely to face similar barriers to education and employment in France. Our approach also classifies second-generation immigrants from North Africa who report that their parents spoke to them only in French and who had a French nationality at birth as children of repatriates. We also believe that this will have a small impact on estimates because studies have shown that while proficiency of Arabic or Berber at adult ages among G2 North African–origin individuals is somewhat variable, exposure to these languages as children in households with two immigrant parents is very high. In the TeO survey, 86% of G2 individuals with two North African immigrant parents reported some exposure to Arabic or Berber when they were children (Condon and Régnard 2016, Annex 5). Moreover, our use of a second variable – nationality at birth – for those reporting that their parents spoke to them only in French at age 5 further alleviates concerns that some second-generation immigrants may be misclassified in our study as children of repatriates.

Figures A-1 and A-2 show that the distinction between immigrants and repatriates is not trivial demographically. Among males, 41% of respondents born in North Africa are identified in the EHF as repatriates and 36% of respondents born in France to two parents born in North Africa are identified as native-born children of repatriates. For females, the proportions are 45% and 39%, respectively.

In this section we examine the impact of making this distinction between immigrants and repatriates from North Africa on our results. First, instead of excluding repatriates from the analysis, as we do in the paper, we treated them as separate G1 and G2 categories, allowing us to examine whether repatriates and their native-born children have a distinct mortality pattern (Table A-11). Second, we estimated our model without making the distinction between immigrants and repatriates from North Africa; that is, we treated all individuals born in North Africa as first-generation immigrants

from North Africa and all individuals born in France to two parents born in North Africa as second-generation immigrants from North Africa (Table A-12).

Results including repatriates as separate G1 and G2 categories (Table A-11) show that for the G2 male subgroups, there is a clear distinction between the children of immigrants per se (G2 North Africa), who as we know exhibit excess mortality, and the children of repatriates (G2 repatriates), who have mortality levels that are not statistically different from the reference population. This is expected given that for the most part children of repatriates from North Africa do not face the same barriers to education and employment as children of immigrants from North Africa per se (Beauchemin, Hamel, and Simon 2016).

Models using country of birth information only for the identification of immigrant subgroups (i.e., without making the distinction between immigrants and repatriates from North Africa) are presented in Table A-12. Results show that when repatriates and immigrants from North Africa are merged, the hazard ratio for G2 North African males decreases from 1.82 to 1.32 and loses significance. This is also expected given the more favorable mortality patterns of G2 repatriates. It illustrates the importance of going beyond parental country of birth information when examining second-generation immigrants from North Africa in the French context.

Table A-11: Mortality hazard ratios (ages 18–64) for first- and second-generation immigrant subgroups by region of origin, France, 1999–2010; G1 and G2 repatriates included as separate subgroup categories

	**Model 1** *(baseline)*						**Model 2** *(+ education level)*					
	**HR**	**SE**	**Sig**	**95% CIs**			**HR**	**SE**	**Sig**	**95% CIs**		
**Males**												
**Generation by region**												
Reference	1						1					
G1												
North Africa	0.79	0.11	†	0.60	–	1.03	0.69	0.09	**	0.53	–	0.90
Repatriates	0.73	0.09	**	0.60	–	0.92	0.79	0.09	*	0.62	–	1.00
G2												
North Africa	1.82	0.39	**	1.20	–	2.78	1.67	0.36	**	1.10	–	2.55
Repatriates	0.75	0.27		0.38	–	1.50	0.76	0.27		0.37	–	1.51
**ISCED education level**												
Tertiary							1					
Secondary			(unadjusted)				1.71	0.10	**	1.53	–	1.91
Primary							2.44	0.15	**	2.17	–	2.74
Missing							2.47	0.25	**	2.02	–	3.02

Table A-11: (Continued)

	Model 1 *(baseline)*						Model 2 *(+ education level)*					
	**HR**	**SE**	**Sig**	**95% CIs**			**HR**	**SE**	**Sig**	**95% CIs**		
**Females**												
**Generation by region**												
Reference	1						1					
G1												
North Africa	1.23	0.23		0.85	–	1.76	1.09	0.20		0.76	–	1.56
Repatriates	1.00	0.15		0.75	–	1.33	1.06	0.15		0.80	–	1.41
G2												
North Africa	0.99	0.38		0.47	–	2.09	0.93	0.35		0.44	–	1.96
Repatriates	1.22	0.43		0.61	–	2.45	1.23	0.44		0.61	–	2.46
**ISCED education level**												
Tertiary							1					
Secondary			(unadjusted)				1.45	0.10	**	1.27	–	1.67
Primary							1.84	0.14	**	1.59	–	2.13
Missing							2.09	0.27	**	1.63	–	2.69

Note: (1) 'reference' refers to individuals born in metropolitan France to two parents born in metropolitan France; (2) significance levels at ** p < 0.01, * p < 0.05, and † p< 0.10.

Table A-12: Mortality hazard ratios (ages 18–64) for first- and second-generation immigrant subgroups by region of origin, France, 1999–2010; North Africa–origin G1 and G2 defined using country of birth information only

	Model 1 (baseline)						Model 2 (+ education level)					
	**HR**	**SE**	**Sig**	**95% CIs**			**HR**	**SE**	**Sig**	**95% CIs**		
**Males**												
**Generation by region**												
Reference	1						1					
G1												
North Africa	0.75	0.07	**	0.63	–	0.90	0.74	0.07	**	0.62	–	0.89
Southern Europe	0.73	0.09	*	0.57	–	0.94	0.61	0.08	**	0.47	–	0.78
Other regions	0.80	0.09	†	0.64	–	1.00	0.84	0.10		0.67	–	1.06
G2												
North Africa	1.32	0.24		0.92	–	1.89	1.27	0.23		0.88	–	1.81
Southern Europe	0.64	0.11	**	0.46	–	0.88	0.62	0.10	**	0.44	–	0.86
Other regions	1.05	0.16		0.78	–	1.42	1.04	0.16		0.77	–	1.40
G2 mixed												
North Africa	0.95	0.16		0.69	–	1.30	0.98	0.16		0.71	–	1.35
Southern Europe	0.81	0.10	†	0.64	–	1.02	0.78	0.09	*	0.61	–	0.99
Other regions	1.01	0.10		0.83	–	1.22	1.04	0.10		0.87	–	1.26
**ISCED education level**												
Tertiary							1					
Secondary			(unadjusted)				1.71	0.10	**	1.53	–	1.91
Primary							2.44	0.15	**	2.17	–	2.74
Missing							2.46	0.25	**	2.01	–	3.00

Table A-12: (Continued)

	**Model 1** (baseline)						**Model 2** (+ education level)					
	**HR**	**SE**	**Sig**	**95% CIs**			**HR**	**SE**	**Sig**	**95% CIs**		
**Females**												
**Generation by region**												
Reference	1						1					
G1												
North Africa	1.08	0.12		0.86	–	1.35	1.06	0.12		0.85	–	1.34
Southern Europe	0.56	0.12	**	0.37	–	0.84	0.49	0.10	**	0.32	–	0.74
Other regions	1.05	0.14		0.81	–	1.36	1.08	0.14		0.84	–	1.41
G2												
North Africa	1.10	0.29		0.66	–	1.83	1.07	0.28		0.64	–	1.78
Southern Europe	0.70	0.15	†	0.46	–	1.06	0.67	0.15	†	0.44	–	1.03
Other regions	0.86	0.19		0.56	–	1.33	0.85	0.19		0.55	–	1.31
G2 mixed												
North Africa	1.11	0.22		0.76	–	1.63	1.14	0.22		0.78	–	1.67
Southern Europe	0.92	0.14		0.69	–	1.24	0.92	0.14		0.68	–	1.23
Other regions	1.33	0.15	**	1.06	–	1.68	1.34	0.15	**	1.07	–	1.68
**ISCED education level**												
Tertiary							1					
Secondary			(unadjusted)				1.45	0.10	**	1.27	–	1.67
Primary							1.87	0.14	**	1.59	–	2.13
Missing							2.09	0.27	**	1.63	–	2.69

Note: (1) 'reference' refers to individuals born in metropolitan France to two parents born in metropolitan France; (2) significance levels at ** p < 0.01, * p < 0.05, and † p< 0.10.

7. Using alternative age breakdowns

The hazard ratios presented in the paper are based on mortality risks for the age range 18 to 64, summarizing mortality at working ages. In this section, we examine whether the hazard ratios for population subgroups vary depending on different age specifications in order to better target ages within the adult age range where subgroups may be particularly vulnerable or advantaged. We focus on the following age groups: 18 to 44 and 45 to 64. These two age groups are distinct epidemiologically, with a larger share of external causes in the age range 18 to 44 and a larger share of noncommunicable diseases in the age range 45 to 64. Results are presented in Table A-13 for mortality at ages 18 to 44 and in Table A-14 for mortality at ages 45 to 64.

The main lesson of this exercise is that excess mortality among second-generation North African–origin males is particularly salient in the age range 18 to 44, with a hazard ratio of 2.02, higher than when considering the entire 18 to 64 age range. No statistically significant excess mortality is detected for this population subgroup at ages 45 to 64. The reverse is true for second-generation southern European–origin males: Their advantage is salient in the age range 45 to 64 but not in the age range 18 to 44.

Results for G2 females, which were not significant in the age range 18 to 64, remain insignificant in these models with alternative age breakdowns.

Table A-13: Mortality hazard ratios (ages 18–44) for first- and second-generation immigrant subgroups by region of origin, France, 1999–2010

	**Model 1** (baseline)						**Model 2** (+ education level)					
	**HR**	**SE**	**Sig**	**95% CIs**			**HR**	**SE**	**Sig**	**95% CIs**		
**Males**												
**Generation by region**												
Reference	1						1					
G1												
North Africa	0.75	0.27		0.37	–	1.50	0.68	0.24		0.34	–	1.36
Southern Europe	1.02	0.34		0.53	–	1.97	0.80	0.27		0.51	–	1.54
Other regions	0.87	0.23		0.52	–	1.44	0.87	0.23		0.52	–	1.45
G2												
North Africa	2.02	0.52	**	1.23	–	3.33	1.74	0.44	*	1.06	–	2.87
Southern Europe	0.78	0.24		0.43	–	1.42	0.73	0.22		0.40	–	1.32
Other regions	0.93	0.42		0.39	–	2.25	0.90	0.40		0.37	–	2.17
G2 mixed												
North Africa	0.77	0.21		0.45	–	1.30	0.79	0.21		0.46	–	1.34
Southern Europe	0.77	0.23		0.42	–	1.39	0.73	0.22		0.40	–	1.32
Other regions	0.71	0.22		0.39	–	1.29	0.73	0.22		0.40	–	1.33
**ISCED education level**												
Tertiary							1					
Secondary	(unadjusted)						2.16	0.28	**	1.67	–	2.80
Primary							3.62	0.54	**	2.70	–	4.84
Missing							4.30	1.01	**	2.72	–	6.81
**Females**												
**Generation by region**												
Reference	1						1					
G1												
North Africa	1.88	0.60	†	1.00	–	3.53	1.52	0.49		0.81	–	2.88
Southern Europe	0.77	0.39		0.29	–	2.08	0.63	0.32		0.24	–	1.70
Other regions	1.47	0.36		0.91	–	2.36	1.42	0.35		0.88	–	2.29
G2												
North Africa	1.31	0.54		0.59	–	2.95	1.18	0.49		0.52	–	2.64
Southern Europe	0.87	0.31		0.43	–	1.75	0.81	0.29		0.40	–	1.64
Other regions	0.81	0.47		0.26	–	2.54	0.77	0.44		0.25	–	2.39
G2 mixed												
North Africa	1.14	0.32		0.65	–	1.98	1.18	0.34		0.68	–	2.06
Southern Europe	0.83	0.29		0.41	–	1.67	0.81	0.29		0.40	–	1.63
Other regions	1.59	0.41	†	0.96	–	2.62	1.62	0.42	†	0.99	–	2.69
**ISCED education level**												
Tertiary							1					
Secondary	(unadjusted)						2.18	0.31	**	1.64	–	2.89
Primary							3.35	0.57	**	2.41	–	4.67
Missing							3.72	1.08	**	2.10	–	6.58

Note: (1) ‘reference’ refers to individuals born in metropolitan France to two parents born in metropolitan France; (2) significance levels at ** p < 0.01, * p < 0.05, and † p< 0.10.

Table A-14: Mortality hazard ratios (ages 45–64) for first- and second-generation immigrant subgroups by region of origin, France, 1999–2010

	Model 1 (baseline)						Model 2 (+ education level)					
	HR	SE	Sig	95% CIs			HR	SE	Sig	95% CIs		
**Males**												
**Generation by region**												
Reference	1						1					
G1												
North Africa	0.83	0.13		0.61	–	1.13	0.71	0.11	*	0.52	-	0.97
Southern Europe	0.69	0.10	*	0.52	–	0.93	0.58	0.09	**	0.44	-	0.78
Other regions	0.80	0.12		0.60	–	1.06	0.84	0.12		0.63	-	1.11
G2												
North Africa	1.02	0.72		0.25	–	4.06	0.97	0.69		0.25	-	3.87
Southern Europe	0.64	0.14	*	0.42	–	0.97	0.63	0.13	*	0.41	-	0.95
Other regions	1.17	0.19		0.85	–	1.62	1.17	0.19		0.84	-	1.62
G2 mixed												
North Africa	1.14	0.27		0.72	–	1.81	1.18	0.28		0.75	-	1.89
Southern Europe	0.85	0.12		0.64	–	1.12	0.82	0.12		0.62	-	1.09
Other regions	0.99	0.11		0.79	–	1.23	1.02	0.12		0.82	-	1.28
**ISCED education level**												
Tertiary							1					
Secondary			(unadjusted)				1.55	0.11	**	1.35	–	1.77
Primary							2.10	0.15	**	1.83	–	2.42
Missing							2.17	0.27	**	1.69	–	2.78
**Females**												
**Generation by region**												
Reference	1						1					
G1												
North Africa	1.10	0.28		0.66	–	1.84	0.97	0.25		0.58	-	1.62
Southern Europe	0.63	0.15	*	0.40	–	0.99	0.55	0.13	*	0.35	-	0.87
Other regions	0.83	0.16		0.57	–	1.22	0.85	0.17		0.58	-	1.25
G2												
North Africa												
Southern Europe	0.81	0.23		0.47	–	1.40	0.79	0.22		0.46	-	1.36
Other regions	1.00	0.24		0.62	–	1.62	0.99	0.24		0.61	-	1.60
G2 mixed												
North Africa	1.05	0.37		0.52	–	2.10	1.07	0.38		0.53	-	2.14
Southern Europe	1.01	0.19		0.70	–	1.45	1.01	0.19		0.70	-	1.45
Other regions	1.42	0.19	**	1.08	–	1.85	1.42	0.20	**	1.08	-	1.86
**ISCED education level**												
Tertiary							1					
Secondary			(unadjusted)				1.19	0.12	†	0.98	–	1.44
Primary							1.47	0.14	**	1.22	–	1.78
Missing							2.01	0.32	**	1.48	–	2.74

Note: (1) 'reference' refers to individuals born in metropolitan France to two parents born in metropolitan France; (2) significance levels at ** p < 0.01, * p < 0.05, and † p< 0.10.
